# Supplementary material for: Nano pom-poms prepared exosomes enable highly specific cancer biomarker detection
Source: Commun Biol. 2022 Jul 4;5:660. doi: 10.1038/s42003-022-03598-0 (PMC9253007; doi:10.1038/s42003-022-03598-0)
Supplement: Supplementary file 2 — Supplementary Information [file 42003_2022_3598_MOESM2_ESM.pdf]

Supplementary Materials

Nano Pom-poms Prepared Exosomes enable Highly Specific Cancer Biomarker Detection

Nan He[1,2], Sirisha Thippabhotla[3], Cuncong Zhong[3], Zachary Greenberg[4], Liang Xu[5], Ziyan Pessetto[6], Andrew K. Godwin[6,7], Yong Zeng[8], Mei He[1,4]*

[1]Department of Chemical and Petroleum Engineering, Bioengineering Program, University of Kansas, Lawrence, KS 66045, USA. [2]Clara Biotech Inc., Lawrence, KS 66047, USA. [3]Department of Electrical Engineering and Computer Science, University of Kansas, Lawrence, KS 66045, USA. [4]Department of Pharmaceutics, College of Pharmacy, University of Florida, Gainesville, FL 32610, USA. [5]Department of Molecular Biosciences, University of Kansas, Lawrence, KS 66045, USA. [6]Department of Pathology and Laboratory Medicine, University of Kansas Medical Center, Kansas City, KS 66160, USA. [7]University of Kansas Cancer Center, Kansas City, KS 66160, USA. [8]Department of Chemistry, University of Florida, Gainesville, FL 32603, USA.
*Correspondence: mhe@cop.ufl.edu.

This PDF file includes:

Supplementary figures and supplementary figure legends (Figs. s1 to s6)
Supplementary tables (Tables s1 to s4)
Supplementary Methods
Supplementary References (1 to 36)

Supplementary figures and supplementary figure legends

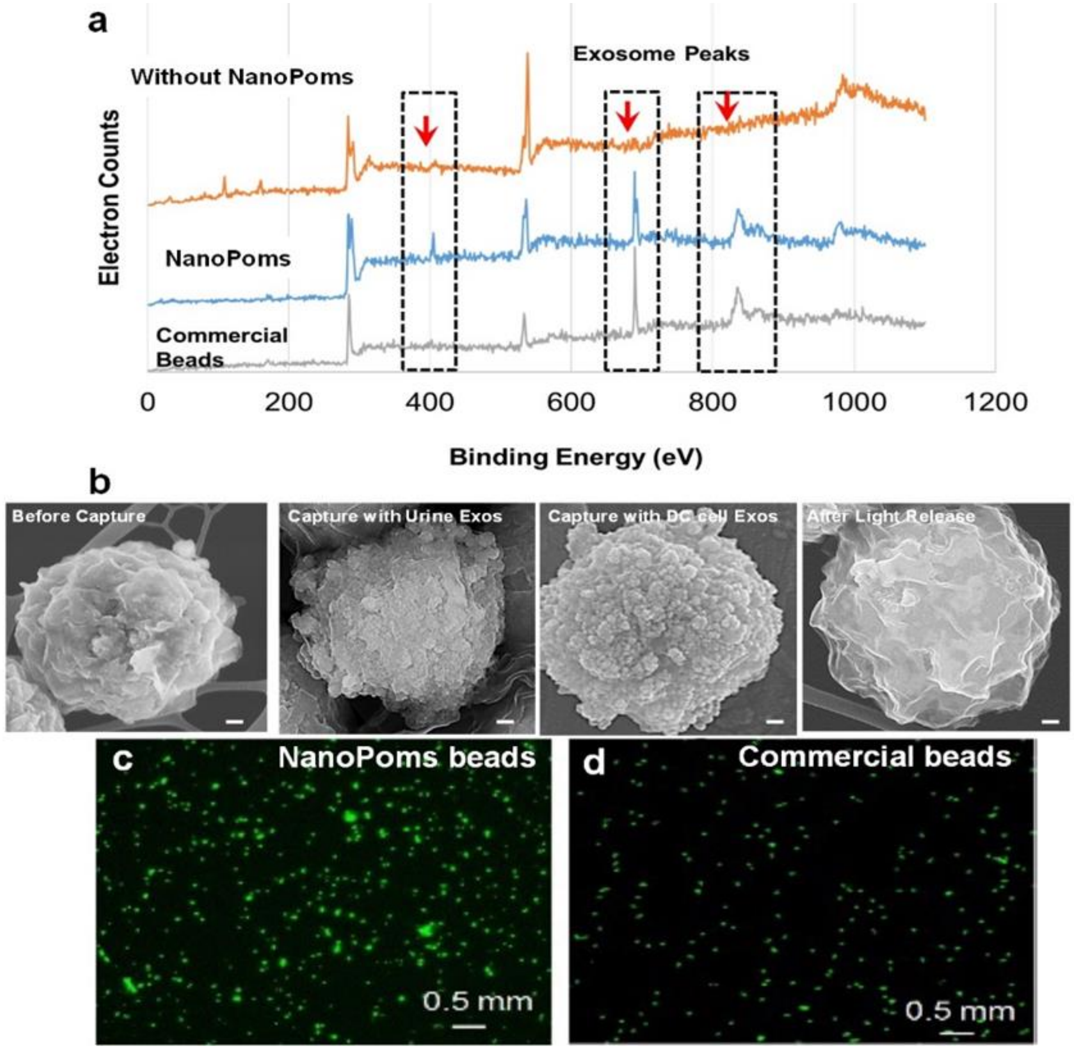

Fig. s1. Characterization of Nano Pom Poms particles for specific capture and release of exosomes.

a) X-Ray Photoelectron Spectroscopy (XPS) analysis of Nano pom-poms surface properties with extracellular vesicles captured. The bare magnetic particles without the 3D-structured nanographene sheet layers serves as the negative control. The commercial dynabeads were used as the positive control. **b)** The SEM images showing the surface morphology of Nano pom-poms before exosome capture, after exosome capture, and after release of captured exosome. The scale bar is 100 nm. The dense round exosomes were seen covering particle surface completely after capture. **c)** Fluorescence microscopic images showing the Nano pom-poms bound to FITC-biotin after conjugation with streptavidin, with dyna streptavidin beads as the positive control **(d)**, which exhibits much brighter fluorescence from Nano pom-poms indicating more binding sites.

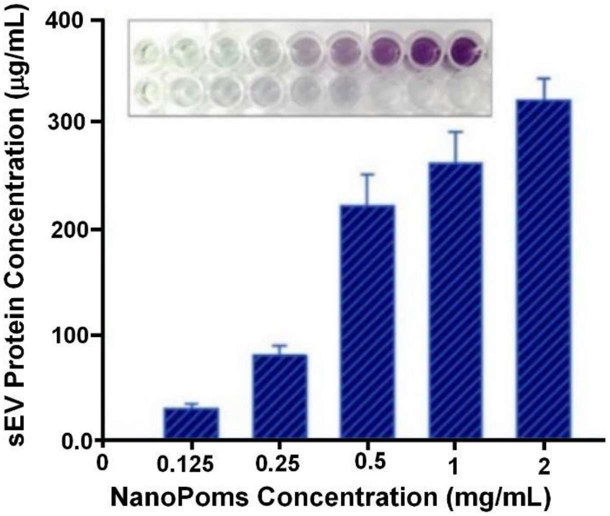

Fig. s2. The optimization of Nano Pom-poms concentration used for isolating exosomes.

The total protein concentration from isolated exosomes is measured by the Pierce BCA Protein Assay. The five repetitive measurements were performed for each data point with RSD < ~5% (n=5). With increasing the amount of capture NanoPoms in 1 mL biofluids (here cow milk), more exosomes were isolated to gradually reach to the maximum. Once the available capture binding sites on the particle surface excess the number of overall exosomes particles, the increase of particle amount does not influence on the overall exosome capture amount. Thus, the 1mg/mL of NanoPoms capture particles were used for appropriate capture efficiency ranging from ~10^8 to ~10^{13} particles/mL.

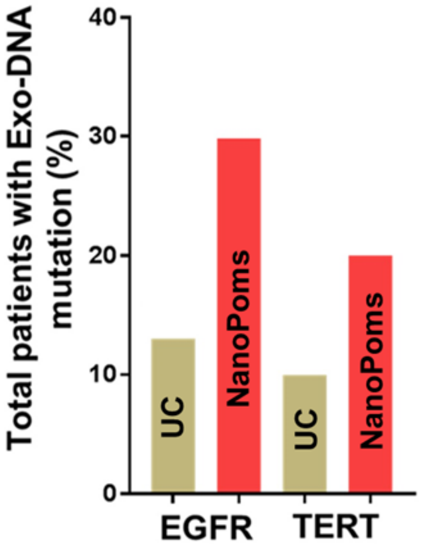

Fig. s3. The droplet digital PCR analysis of NanoPoms prepared exosomes with substantially improved detection specificity and sensitivity.

The ddPCR analysis of the mutation frequency *EGFR* (Thr790Met) and *TERT* (C228T and C250T) using DNAs extracted from either NanoPoms or UC prepared urinary exosomes. The 30 bladder cancer patient urine samples were used. The same amount of exosome DNAs (10 µg) were used as the sample input. The detection efficiency from NanoPoms was 3-fold higher as compared to UC approach.

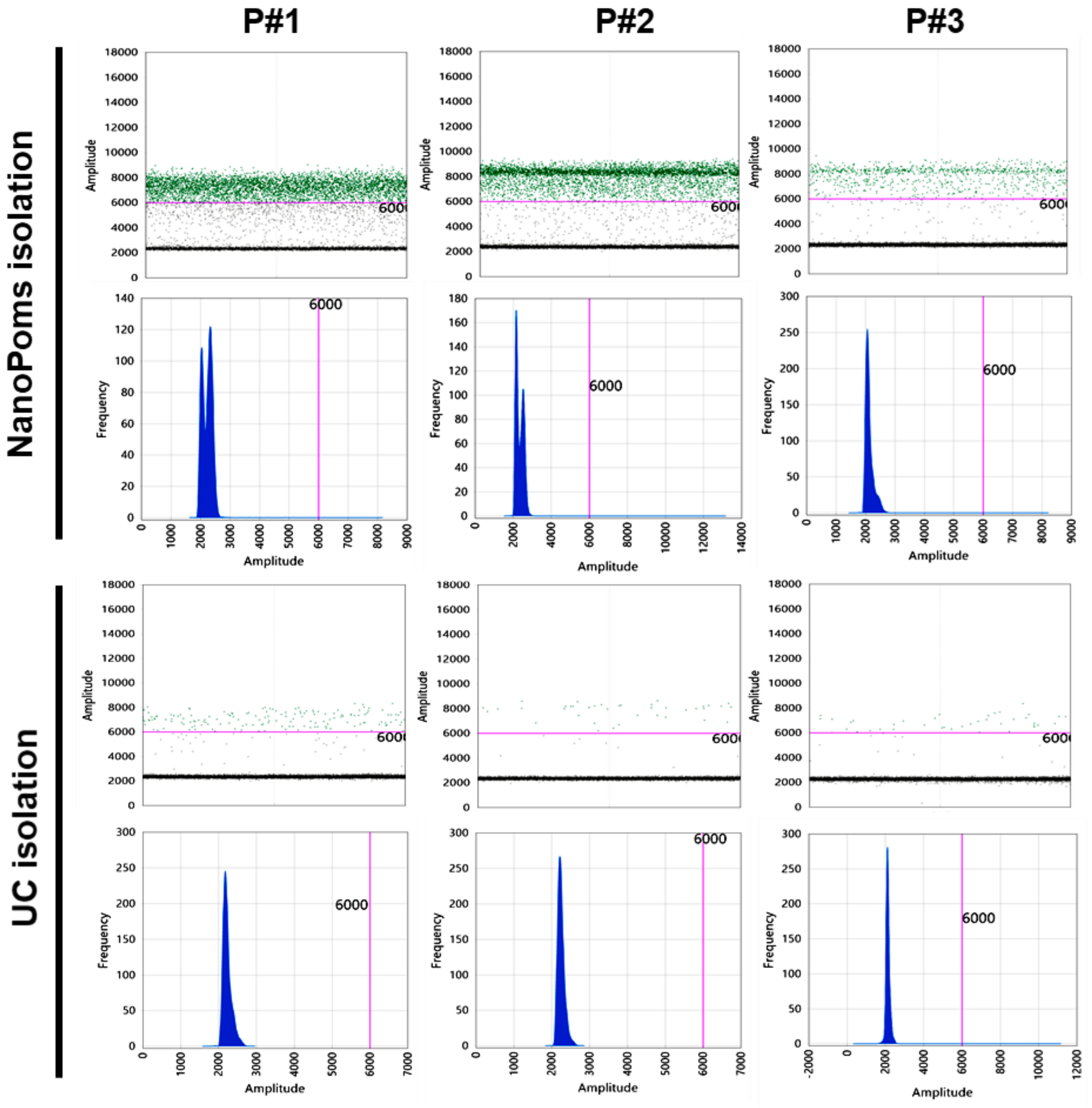

Fig. s4. The droplet digital PCR analysis of NanoPoms prepared exosomes detects the EGFR heterozygosity.

The EGFR heterozygosity from three bladder cancer patient urine samples was detected using NanoPoms exosome preparation. In contrast, UC isolated exosome DNAs from the same sample input did not lead to the detection of EGFR heterozygosity in patient 2 and patient 3. The DNA copy numbers were substantially lower in UC prepared exosome DNAs as compared to NanoPoms.

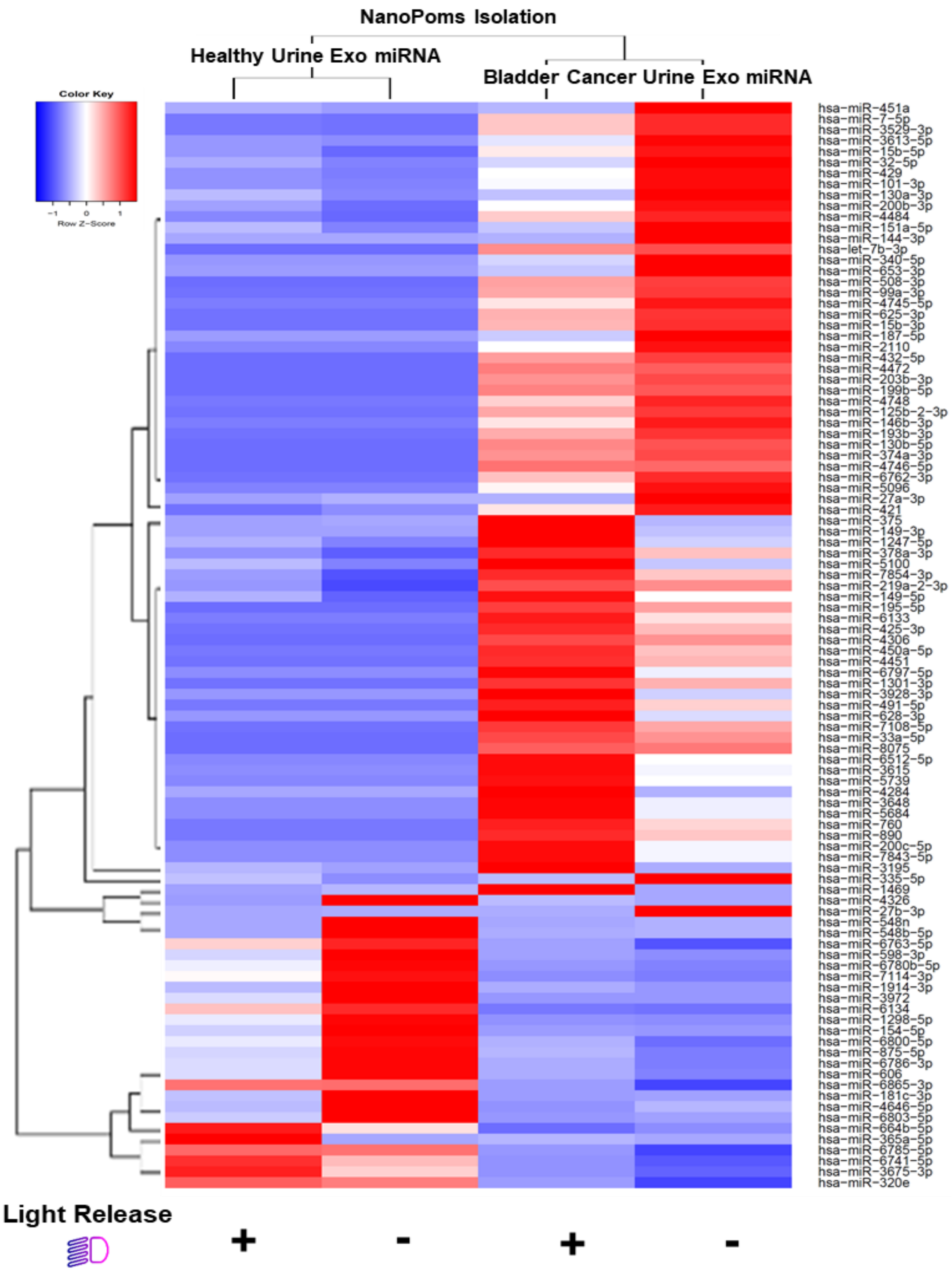

Fig. s5. Heatmap dendrogram clustering analysis depicts the top 100 highly expressed miRNAs from urinary exosomes derived from both the BC patient and the healthy control. The small RNAs were extracted from NanoPoms exosomes preparation with and without light release process. Red color indicates a higher expression z-score. Hierarchical clustering was performed, using the Spearman correlation method. The disease group can be significantly differentiated from healthy group regardless whether the light release process was implemented.

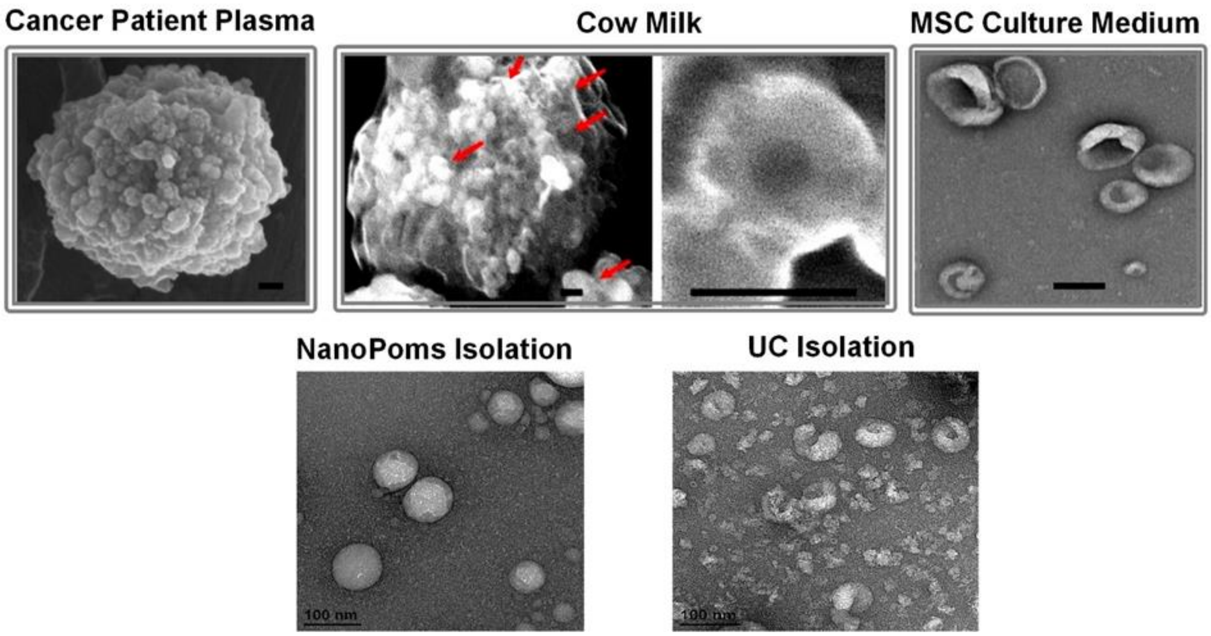

Fig. s6. The SEM and TEM morphological characterization of exosome isolated from a variety of biological fluids using Nano pom-poms.

Top: The SEM images showing the morphology of Nano pom-poms captured exosomes from ovarian cancer patient plasma (left), cow's milk with enlarged insert showing the classic cup shape (middle), released from the Wharton's jelly mesenchymal stem cell culture medium (right). The scale bar indicates the 100 nm. Bottom: TEM images showing the much clean and uniform exosome isolated from NanoPoms preparation from cell culture medium. In contrast, ultracentrifugation prepares exosomes in a mixture with small aggregates and debris.

Raw images of Western Blotting analysis in Figure 5a

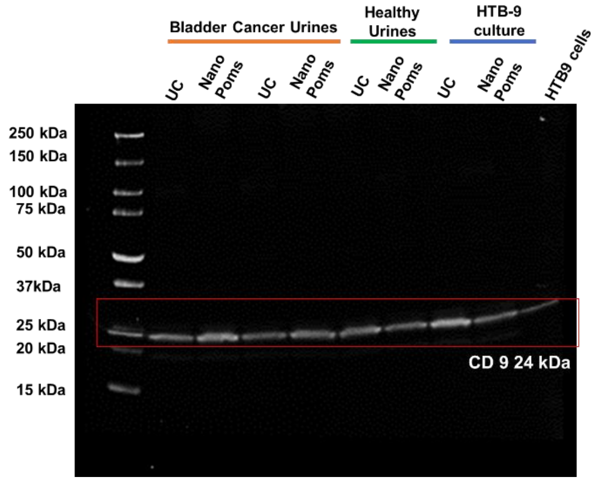

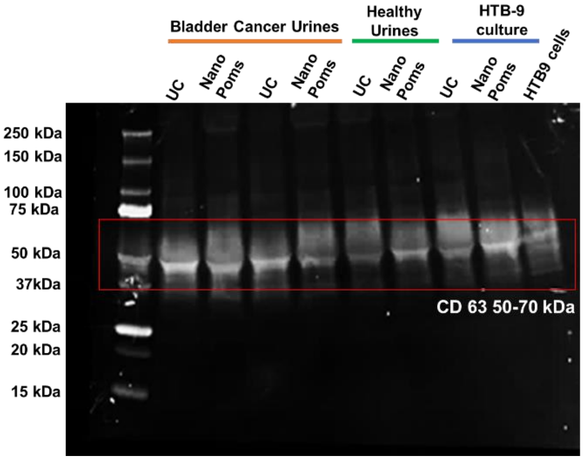

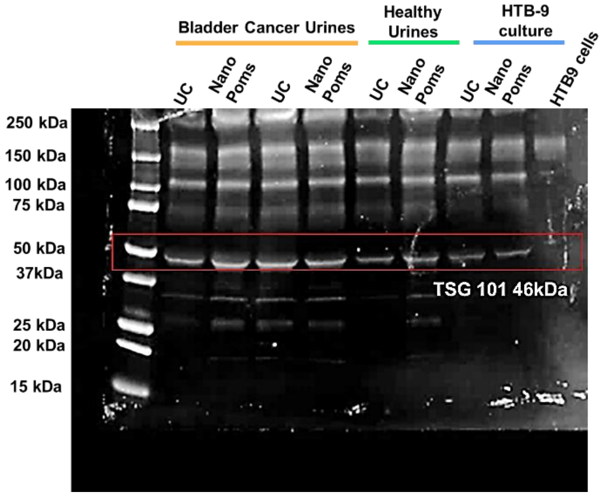

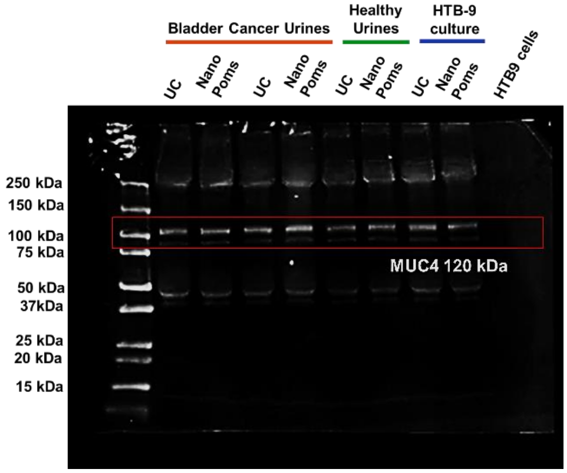

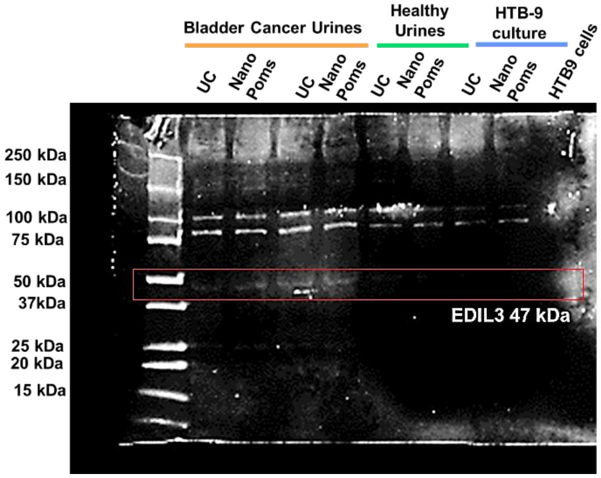

Supplementary tables

Table s1.

The distribution of small RNAs from urinary exosome isolated by UC and NanoPoms.

%	***tRNA***	***lncRNA***	***miRNA***	***snRNA***	***snoRNA***	***miscRNA***	***rRNA***	***scaRNA***	***Mt-tRNA***	***Mt_rRNA***	***scRNA***	***vaultRNA***	***sRNA***	***Other***
UC-BC Urine Exosomes	64.35	18.89	12.40	1.92	0.18	0.50	1.39	0.01	0.02	0.002	0.00	0.004	0.001	0.34
NanPoms-BC Urine Exosomes	47.52	42.02	4.94	1.41	0.29	0.89	1.98	0.07	0.03	0.01	0.00	0.002	0.001	0.82
UC-Healthy Urine Exosomes	69.88	14.68	8.59	4.66	0.23	0.46	1.09	0.01	0.003	0.003	0.00	0.003	0.001	0.38
NanoPoms-Healthy Urine Exosomes	49.62	42.10	4.19	1.16	0.38	0.51	0.85	0.01	0.03	0.01	0.00	0.002	0.001	1.14

Table s2.

The top 10 highly enriched miRNAs identified from NanoPoms isolated urinary exosomes as compared with UC.

Genes	*Log2 Fold Change*	*p value*	*Reported signaling pathway/functions*	*Ref*
hsa-miR-3168	5.140358266	1.38E-07	KRAS-dependent sorting to exosomes from colorectal cancer cell lines<br><br>Known Melanoma mature miRNA	1, 2
hsa-miR-92b-5p	4.358349687	1.08E-06	Cancer metastasis<br><br>Promote EMT in bladder cancer migration	3, 4
hsa-miR-891a-5p	-5.329381923	1.19E-06	Prognostic marker for HR-positive breast cancer	5
hsa-miR-6785-5p	5.063745282	1.80E-06	miRNA target genes in the TP53 signaling pathway in tumor	6
hsa-miR-934	-4.078662275	3.10E-06	Cancer metastasis, exosomal oncogene<br><br>Non-coding RNA with neurogenic function	7-9
hsa-miR-6883-3p	4.420682437	5.64E-06	Target CDK4/6 in colon cancer cells	10
hsa-miR-3202	5.039882582	8.31E-06	Regulating TLR signaling pathway<br><br>Promoted Cell Apoptosis	11, 12
hsa-miR-3648	-4.682609314	1.30E-05	Promote invasion and metastasis of human bladder cancer<br><br>Regulate cell proliferation	13, 14
hsa-miR-6802-5p	4.168335059	1.84E-05	Exosomal regulatory miRANs<br><br>Heart disease	15, 16
hsa-miR-6763-5p	4.0778099	2.21E-05	Immunity Regulation	17, 18

Table s3.

The 11 unique gene products identified from BC patient only and 4 unique genes identified from healthy control group only, by proteomic analysis of NanoPoms isolated urinary exosome proteins. The Human Protein Atlas database was used: https://www.proteinatlas.org/

BC Patient	*FASTA Title Lines*	*Reported Functions and Pathways*
ARMCX4	|Q5H9R4|ARMX4_HUMAN Armadillo repeat-containing X-linked protein 4	Intracellular, Nucleoplasm and additionally in Vesicles<br>Prognostic marker, novel passenger cancer genes[19, 20]
DSC3	|Q14574|DSC3_HUMAN Desmocollin-3	Plasma membrane, Cell Junctions<br>Regulated by p53 signaling pathway in colorectal cancer[21]<br>Down-regulated in primary breast tumors[22]
IRAK4	|Q9NWZ3|IRAK4_HUMAN Interleukin-1 receptor-associated kinase 4	Intracellular, Microtubules and additionally in Nucleoli, Cytosol<br>Prognostic marker in endometrial cancer and urothelial cancer<br>Disrupts inflammatory pathways and delays tumor development[23, 24]
KRT23	|Q9C075|K1C23_HUMAN Keratin, type I cytoskeletal 23	Intracellular, Intermediate filaments and additionally in Cytosol<br>Prognostic marker in urothelial cancer<br>Keratin 23 promotes telomerase reverse transcriptase expression and human colorectal cancer growth[25, 26]
PIGQ	|Q9BRB3|PIGQ_HUMAN Phosphatidylinositol N-acetylglucosaminyltransferase subunit Q	Golgi apparatus, Vesicles and additionally in Nucleoplasm<br>Prognostic marker in renal cancer<br>GPI-AP biosynthesis deficiency disorder syndrome[27]
SERPINB2	|P05120|PAI2_HUMAN Plasminogen activator inhibitor 2	Intracellular<br>Prognostic marker in urothelial cancer<br>SerpinB2 inhibits migration and promotes a resolution phase signature in large peritoneal macrophages[28]
PDHA2	|P29803|ODPAT_HUMAN Pyruvate dehydrogenase E1 component subunit alpha, testis-specific form, mitochondrial	Intracellular, Mitochondria, links the glycolytic pathway to the tricarboxylic cycle<br>Testis specific[29]<br>In Tumor Suppressor gene database https://bioinfo.uth.edu/TSGene/,
RALGAPA2	|Q2PPJ7|RGPA2_HUMAN Ral GTPase-activating protein subunit alpha-2	Intracellular, Plasma membrane, Cytosol<br>Prognostic marker in renal cancer<br>Downregulation of Ral GTPase-activating protein promotes tumor invasion and metastasis of bladder cancer[30]
SMARCD3	|Q6STE5|SMRD3_HUMAN SWI/SNF-related matrix-associated actin-dependent regulator of chromatin subfamily D member 3	Intracellular, Nucleoplasm<br>Prognostic marker in colorectal cancer<br>The chromatin remodeler SMARCD3 regulates cell cycle progression and its expression predicts survival outcome in ER+ breast cancer[31, 32]
PAPD7	|Q5XG87|PAPD7_HUMAN Non-canonical poly(A) RNA polymerase	Intracellular, Nucleoplasm and additionally in Nuclear membrane, Golgi apparatus<br>Prognostic marker in renal cancer and urothelial cancer
HIST1H2BO	HIST1H2BO_HUMAN Histone H2B type 1-O	Intracellular, Located in Nucleoplasm, Cytosol<br>Prognostic marker in urothelial cancer
Healthy	***FASTA Title Lines***	***Reported Functions and Pathways***
ORM2	|P19652|A1AG2_HUMAN Alpha-1-acid glycoprotein 2	Intracellular, Vesicles and additionally in Golgi apparatus<br>Orm1 and Orm2 are conserved endoplasmic reticulum membrane proteins regulating lipid homeostasis and protein quality control[33-35]
ATP5F1A	|P25705|ATPA_HUMAN ATP synthase subunit alpha, mitochondrial	Intracellular, Mitochondria<br>Reduced Levels of ATP Synthase Subunit ATP5F1A Correlate with Earlier-Onset Prostate Cancer[36]
DEFB1	|P60022|DEFB1_HUMAN Beta-defensin 1	Secreted pathway<br>Normal tissue annotation
MPP7	|Q5T2T1|MPP7_HUMAN MAGUK p55 subfamily member 7	Intracellular, Cell Junctions and additionally in Nucleoplasm<br>Acts as an important adapter that promotes epithelial cell polarity and tight junction formation via its interaction with DLG1. Involved in the assembly of protein complexes at sites of cell-cell contact

Table s4.

Comparative analysis of cost, time, steps, and performance between exosome isolation methods.

**Exosome Isolation Methods**	**NanoPoms**	**Ultracentrifugation**	**Chromatographic Column**	**Polymer Precipitation**
**Cost**	Low, no instrument required	High, very expensive equipment cost	Low	Low
**Protocol duration**	~4 hrs	~12 hrs	~6 hrs	~6 hrs
**Processing capacity**	From ~ µL to L	~12- 500 mL	~ 150 µL-100 mL (IZON)	~ ≤ 1 mL ExoQuick™
**Subtype specificity**	Surface marker defined specificity to subtypes	No	No	No
**Purity**	High to homogeneous subpopulations	Mixture of EV populations	Mixture of EV populations	Mixture of EV with protein aggregates
**Reproducibility**	High	Low	-	-
**Scalability**	Yes	No	Yes	No

Supplementary Methods

NGS analysis of urinary exosome small RNAs

Bioinformatics Analysis: The sequences of precursor miRNA, tRNA, snRNA, snoRNA, scaRNA, rRNA, scRNA, vaultRNA, lncRNA, miscRNA, pseudogenes, retained introns, ribozymes and transcribed unprocessed pseudogenes were extracted from RefSeq (hg38) and GENCODE (v29) to build a customized database. We refer to this database as the customized ncRNA database. For further quantifying mature miRNA abundance, their sequences were extracted from miRBase1 (ver. hg38) to form another database, which is refer to as the mature miRNA database. Initial quality assessment of the reads was performed using the FASTQC (https://www.bioinformatics.babraham.ac.uk/projects/fastqc/) package. Cutadapt was used to trim the 5' (GUUCAGAGUUCUACAGUCCGACGAUC) and 3' (AACTGTAGGCACCATCAAT) adaptor sequences from the sequences. The unique molecular indices (UMI) sequences in the QIAseq libraries were further trimmed using FASTP (--umi_loc=read1 --umi_len=12). The trimmed reads were filtered with a minimum length of 15nt. Mapping to the mature miRNA database and customized non-ncRNA database was performed using BWA (bwa-aln)2. We relaxed the BWA parameter to allow each read to mapped to at most 100 locations, accounting for the numerous multi-mapping cases induced by the short-read length of the sequencing reads. To ensure maximum accuracy, we further restrict perfect sequence in seeding (by setting -k=0). All other parameters were used as default. With an in-house script, we counted the read mapping to quantify the expression level of each ncRNA gene and the abundance of each mature miRNA. For multi-mapping, we evenly distributed its abundances to all mapped locations. The mapping results against the customized ncRNA database were used to quantify different types of ncRNAs as the Pie charts (refer pie-chart figures here). The mapping results against the mature miRNA database were summarized as the volcano plot (refer volcano plot here). The mapping results were further analyzed using the Deseq2 3 to reveal significantly different abundant miRNAs. The most significant 100 miRNAs were further selected to generate the heatmap (shown below).

Proteomic analysis of urinary exosome proteins

Urinary exosome pellets resultant from ~2 mL of urine from both bladder cancer patients and healthy individuals (four biological replications for each group) were reconstituted in 400 µL of M-PER Mammalian Protein Extraction Buffer (Thermo) supplemented with 1× Halt Protease Inhibitors (Thermo) and sonicated in an ultrasonic water bath for 15 min. NanoPom exosome samples were processed by in-gel digestion for LC-MS analysis. Approximately 20 µg of protein from each sample was resolved on a 4-20% gradient gel (Bio-Rad), then visualized by SimplyBlue SafeStain (ThermoFisher). Each lane was cut into 8 slices of approximately the same size, then reduced, alkylated, and digested with 400 ng of trypsin overnight at 37°C. Digestion was quenched with 1% formic acid (FA) in 50 mM ammonium bicarbonate buffer/50% acetonitrile (ACN). Peptides were dried using a speed-vac and stored at -20°C. For LC-MS analysis, peptides were reconstituted in 3% ACN/0.1% FA. Peptides were injected onto an Acclaim PepMap 100 C18 trap column (75 µm x 2 cm, ThermoFisher) using an Agilent 1260 Infinity capillary pump and auto sampler (Agilent Technologies). The autosampler was maintained at 4°C, the capillary pump flow rate was set to 1.5 µL/min, and an isocratic solvent system consisting of 3% ACN/0.1% FA. After 10 minutes, the trap column was valve was switched to be in-line with an Acclaim PepMap RSLC

C18 analytical column (50 µm x 25 cm, ThermoFisher), using an Agilent 1290 Infinity II column compartment, kept at 42°C. Peptides were resolved on the analytical column using an Agilent 1290 Infinity II UHPLC with nanoflow passive split flow adapter, maintaining 200 µL/min flow pre-split, resulting in ~300 nL/min flow on the analytical column at the beginning of the run. A two solvent system consisting of (A) water/0.1% FA and (B) ACN/0.1% FA was used, with a gradient as follows: 3% B at 0 min, ramping to 8% B at 8 minutes, ramping to 26% B at 90 min, ramping to 35% B at 105 min, ramping to 40% B at 120 min, then ramping to 70% B at 122, held at 70% B until 127 min, before returning to 3% at 130 min and holding until the end of the run at 150 min, with a post run equilibration of 12 min. Eluted peptides were analyzed by an Agilent 6550 QToF mass spectrometer equipped with a G1992A nanoESI source (Agilent Technologies). The source parameters were as follows: drying gas temperature was set to 200°C, flow of 11 L/min, a capillary voltage of 1200 V, and fragmentor voltage of 360 V was used. Data was acquired in positive ion mode using data dependent acquisition, with an MS scan range of 290 to 1700 m/z at 8 spectra/s, MS/MS scan range of 50-1700 m/z at 3 spectra/s, and an isolation width set to narrow (~1.3 m/z). Maximum precursors per cycle was set to 10, with dynamic exclusion enabled after 2 spectra, and released time set to 0.5 min. Peptides were fragmented by collision induced dissociation (CID) using N2 gas and a variable collision energy depending on the precursor charge and m/z. Reference mass correction in real time was enabled, with lock masses of 299 and 1221 m/z used. Data acquired for each sample was converted to Mascot Generic Format (.MGF) using the Agilent Data Reprocessor (Agilent Technologies). Database searching of .MGF files was done using Mascot Daemon v2.2.2 (Matrix Science). Data was searched against a concatenated decoy FASTA file containing Homo sapiens proteins downloaded from Uniprot. Search results from all 3 engines was combined and analyzed using Scaffold4 v4.8.1 (Proteome Software Inc.). Thresholds of 1% FDR protein, 1% FDR peptide, and 2 peptides minimum were set for protein identification.

Supplementary References

1. Stark, M.S. et al. Characterization of the Melanoma miRNAome by Deep Sequencing. *PLoS One* **5**, e9685 (2010).
2. Cha, D.J. et al. KRAS-dependent sorting of miRNA to exosomes. *Elife* **4**, e07197 (2015).
3. Ma, G. et al. Integrin alpha6 promotes esophageal cancer metastasis and is targeted by miR-92b. *Oncotarget* **8**, 6681-6690 (2017).
4. Huang, J. et al. miR-92b targets DAB2IP to promote EMT in bladder cancer migration and invasion. *Oncol Rep* **36**, 1693-1701 (2016).
5. Zhang, Z. et al. MiR-891a-5p as a prognostic marker and therapeutic target for hormone receptor-positive breast cancer. *J Cancer* **11**, 3771-3782 (2020).
6. He, C. et al. Comprehensive bioinformatics analysis of the TP53 signaling pathway in Wilms' tumor. *Ann Transl Med* **8**, 1228 (2020).
7. Prodromidou, K. et al. MicroRNA-934 is a novel primate-specific small non-coding RNA with neurogenic function during early development. *Elife* **9** (2020).
8. Zhao, S. et al. Tumor-derived exosomal miR-934 induces macrophage M2 polarization to promote liver metastasis of colorectal cancer. *J Hematol Oncol* **13**, 156 (2020).
9. Hu, Y. et al. Oncogene miR-934 promotes ovarian cancer cell proliferation and inhibits cell apoptosis through targeting BRMS1L. *Eur Rev Med Pharmacol Sci* **23**, 5595-5602 (2019).
10. Lulla, A.R. et al. miR-6883 Family miRNAs Target CDK4/6 to Induce G1 Phase Cell-Cycle Arrest in Colon Cancer Cells. *Cancer Res* **77**, 6902-6913 (2017).

11. Chen, J., Liu, Z. & Yang, Y. In vitro screening of LPS-induced miRNAs in leukocytes derived from cord blood and their possible roles in regulating TLR signals. *Pediatr Res* **75**, 595-602 (2014).
12. Huang, X., Xie, H., Xue, G., Ye, M. & Zhang, L. MiR-3202 - Promoted H5V Cell Apoptosis by Directly Targeting Fas Apoptotic Inhibitory Molecule 2 (FAIM2) in High Glucose Condition. *Med Sci Monit* **23**, 975-983 (2017).
13. Sun, W. et al. MicroRNA-3648 Is Upregulated to Suppress TCF21, Resulting in Promotion of Invasion and Metastasis of Human Bladder Cancer. *Mol Ther Nucleic Acids* **16**, 519-530 (2019).
14. Rashid, F., Awan, H.M., Shah, A., Chen, L. & Shan, G. Induction of miR-3648 Upon ER Stress and Its Regulatory Role in Cell Proliferation. *Int J Mol Sci* **18** (2017).
15. Liu, Y. et al. Role of miRNAs in Epicardial Adipose Tissue in CAD Patients with T2DM. *Biomed Res Int* **2016**, 1629236 (2016).
16. Yan, S. et al. Altered microRNA profiles in plasma exosomes from mesial temporal lobe epilepsy with hippocampal sclerosis. *Oncotarget* **8**, 4136-4146 (2017).
17. Wang, C. et al. MicroRNA expression profile of HCT-8 cells in the early phase of Cryptosporidium parvum infection. *BMC Genomics* **20**, 37 (2019).
18. Migita, K. et al. Circulating microRNA Profiles in Patients with Type-1 Autoimmune Hepatitis. *PLoS One* **10**, e0136908 (2015).
19. Chang, Y.S., Huang, H.D., Yeh, K.T. & Chang, J.G. Identification of novel mutations in endometrial cancer patients by whole-exome sequencing. *Int J Oncol* **50**, 1778-1784 (2017).
20. Choi, S.M. et al. KRAS mutation in secondary malignant histiocytosis arising from low grade follicular lymphoma. *Diagn Pathol* **13**, 78 (2018).
21. Cui, T. et al. DSC3 expression is regulated by p53, and methylation of DSC3 DNA is a prognostic marker in human colorectal cancer. *Br J Cancer* **104**, 1013-1019 (2011).
22. Oshiro, M.M. et al. Epigenetic silencing of DSC3 is a common event in human breast cancer. *Breast Cancer Res* **7**, R669-680 (2005).
23. Gimenez, N. et al. Targeting IRAK4 disrupts inflammatory pathways and delays tumor development in chronic lymphocytic leukemia. *Leukemia* **34**, 100-114 (2020).
24. Corzo, C.A. et al. The kinase IRAK4 promotes endosomal TLR and immune complex signaling in B cells and plasmacytoid dendritic cells. *Sci Signal* **13** (2020).
25. Zhang, N. et al. Keratin 23 promotes telomerase reverse transcriptase expression and human colorectal cancer growth. *Cell Death Dis* **8**, e2961 (2017).
26. Kim, D. et al. Keratin 23 Is a Peroxisome Proliferator-Activated Receptor Alpha-Dependent, MYC-Amplified Oncogene That Promotes Hepatocyte Proliferation. *Hepatology* **70**, 154-167 (2019).
27. Starr, L.J., Spranger, J.W., Rao, V.K., Lutz, R. & Yetman, A.T. PIGQ glycosylphosphatidylinositol-anchored protein deficiency: Characterizing the phenotype. *Am J Med Genet A* **179**, 1270-1275 (2019).
28. Schroder, W.A. et al. SerpinB2 inhibits migration and promotes a resolution phase signature in large peritoneal macrophages. *Sci Rep* **9**, 12421 (2019).
29. Pinheiro, A. et al. Demethylation of the coding region triggers the activation of the human testis-specific PDHA2 gene in somatic tissues. *PLoS One* **7**, e38076 (2012).
30. Saito, R. et al. Downregulation of Ral GTPase-activating protein promotes tumor invasion and metastasis of bladder cancer. *Oncogene* **32**, 894-902 (2013).

31. Tropee, R. et al. The SWI/SNF subunit SMARCD3 regulates cell cycle progression and predicts survival outcome in ER+ breast cancer. *Breast Cancer Res Treat* (2020).
32. Jiang, M., Wang, H., Chen, H. & Han, Y. SMARCD3 is a potential prognostic marker and therapeutic target in CAFs. *Aging (Albany NY)* **12**, 20835-20861 (2020).
33. Han, S., Lone, M.A., Schneiter, R. & Chang, A. Orm1 and Orm2 are conserved endoplasmic reticulum membrane proteins regulating lipid homeostasis and protein quality control. *Proc Natl Acad Sci U S A* **107**, 5851-5856 (2010).
34. Zhang, X. et al. The potential role of ORM2 in the development of colorectal cancer. *PLoS One* **7**, e31868 (2012).
35. Gao, F., Zhang, X., Whang, S. & Zheng, C. Prognostic impact of plasma ORM2 levels in patients with stage II colorectal cancer. *Ann Clin Lab Sci* **44**, 388-393 (2014).
36. Feichtinger, R.G. et al. Reduced Levels of ATP Synthase Subunit ATP5F1A Correlate with Earlier-Onset Prostate Cancer. *Oxid Med Cell Longev* **2018**, 1347174 (2018).
