# Supplementary material for: Nano pom-poms prepared exosomes enable highly specific cancer biomarker detection
Source: Commun Biol. 2022 Jul 4;5:660. doi: 10.1038/s42003-022-03598-0 (PMC9253007; doi:10.1038/s42003-022-03598-0)
Supplement: Supplementary file 3 — Description of Additional Supplementary Files [file 42003_2022_3598_MOESM3_ESM.pdf]

Description of Additional Supplementary Files

File name: Supplementary Data 1
Description: Source data presented in the figures.
