# Supplementary material for: Nano pom-poms prepared exosomes enable highly specific cancer biomarker detection
Source: Commun Biol. 2022 Jul 4;5:660. doi: 10.1038/s42003-022-03598-0 (PMC9253007; doi:10.1038/s42003-022-03598-0)
Supplement: Supplementary file 5 — Reporting Summary [file 42003_2022_3598_MOESM5_ESM.pdf]

nature portfolio

Corresponding author(s): Mei He, Ph.D., University of Florida

Last updated by author(s): 01/19/2022

Reporting Summary

Nature Portfolio wishes to improve the reproducibility of the work that we publish. This form provides structure for consistency and transparency in reporting. For further information on Nature Portfolio policies, see our Editorial Policies and the Editorial Policy Checklist.

Statistics

For all statistical analyses, confirm that the following items are present in the figure legend, table legend, main text, or Methods section.

n/a	Confirmed	
☐	☒	The exact sample size (*n*) for each experimental group/condition, given as a discrete number and unit of measurement
☐	☒	A statement on whether measurements were taken from distinct samples or whether the same sample was measured repeatedly
☐	☒	The statistical test(s) used AND whether they are one- or two-sided<br>*Only common tests should be described solely by name; describe more complex techniques in the Methods section.*
☐	☒	A description of all covariates tested
☒	☐	A description of any assumptions or corrections, such as tests of normality and adjustment for multiple comparisons
☐	☒	A full description of the statistical parameters including central tendency (e.g. means) or other basic estimates (e.g. regression coefficient) AND variation (e.g. standard deviation) or associated estimates of uncertainty (e.g. confidence intervals)
☐	☒	For null hypothesis testing, the test statistic (e.g. *F*, *t*, *r*) with confidence intervals, effect sizes, degrees of freedom and *P* value noted<br>*Give P values as exact values whenever suitable.*
☒	☐	For Bayesian analysis, information on the choice of priors and Markov chain Monte Carlo settings
☒	☐	For hierarchical and complex designs, identification of the appropriate level for tests and full reporting of outcomes
☒	☐	Estimates of effect sizes (e.g. Cohen's *d*, Pearson's *r*), indicating how they were calculated

Our web collection on statistics for biologists contains articles on many of the points above.

Software and code

Policy information about availability of computer code

Data collection	n/a
Data analysis	All statistical tests were performed under the open-source statistics using GraphPad Prism software 8 (San Diego, CA), including heatmap, ROC analysis, and clustering analysis. The one-way ANOVA and t test were used. Differences are considered statistically significant at P < 0.05. *P < 0.05; **P < 0.01; ***P < 0.001. The Venn diagram was analyzed using open software from the Bioinformatics & Evolutionary Genomics. The bioinformatic analysis of small RNAs was detailed in supplementary material.

For manuscripts utilizing custom algorithms or software that are central to the research but not yet described in published literature, software must be made available to editors and reviewers. We strongly encourage code deposition in a community repository (e.g. GitHub). See the Nature Portfolio guidelines for submitting code & software for further information.

Data

Policy information about availability of data

All manuscripts must include a data availability statement. This statement should provide the following information, where applicable:

- Accession codes, unique identifiers, or web links for publicly available datasets
- A description of any restrictions on data availability
- For clinical datasets or third party data, please ensure that the statement adheres to our policy

All data generated or analysed during this study are included in this published article (and its supplementary information files). The datasets generated during and/or analysed during the current study are available from the corresponding author on reasonable request.

Field-specific reporting

Please select the one below that is the best fit for your research. If you are not sure, read the appropriate sections before making your selection.

☒ Life sciences ☐ Behavioural & social sciences ☐ Ecological, evolutionary & environmental sciences

For a reference copy of the document with all sections, see nature.com/documents/nr-reporting-summary-flat.pdf

Life sciences study design

All studies must disclose on these points even when the disclosure is negative.

Sample size	For proofing concept of technology platform, our work collected sufficient data sample size (n>3) to draw statistically reliable conclusions. For future to proof the clinical potential and significance, the power analysis will be used to justify patient sample size which is out of the scope of current manuscript.
Data exclusions	n/a
Replication	For particle tracking analysis (NTA), five measures were conducted to obtain the average value shown in Figure 1D. Four sample replications were used in Figure 1E . For Figure 4 and 5, due to the limited sample volume from matched patients for validating different analytical methods (RNA sequencing, western blotting, and proteomic mass spectrometry), there is no replication of samples.
Randomization	n/a
Blinding	n/a

Reporting for specific materials, systems and methods

We require information from authors about some types of materials, experimental systems and methods used in many studies. Here, indicate whether each material, system or method listed is relevant to your study. If you are not sure if a list item applies to your research, read the appropriate section before selecting a response.

Materials & experimental systems

n/a	Involved in the study	
☐	☒	Antibodies
☒	☐	Eukaryotic cell lines
☒	☐	Palaeontology and archaeology
☐	☒	Animals and other organisms
☒	☐	Human research participants
☒	☐	Clinical data
☒	☐	Dual use research of concern

Methods

n/a	Involved in the study	
☒	☐	ChIP-seq
☒	☐	Flow cytometry
☒	☐	MRI-based neuroimaging

Antibodies

Antibodies used	anti-CD9 (Thermo Fisher, 10626D), anti-CD63 (Thermo Fisher, 10626D), Anti- CD81 (Ancell, 302-030), anti-EDIL3 (Abcam, ab88667), anti-MUC4 (Abcam, ab60720), anti-TSG101 (Invitrogen, PA5-86445)
Validation	antibodies have been validated from their corresponding websites using WB, IHC-P and Flow Cyt. Data is shown from product websites from Thermo Fisher, Abcam, Ancell, and Invitrogen.

Animals and other organisms

Policy information about studies involving animals; ARRIVE guidelines recommended for reporting animal research

Laboratory animals	The 6- to 8-week-old female BALB/cJ mice were used. T
Wild animals	Did not involve wide animals
Field-collected samples	Did not involve field collected samples
Ethics oversight	he animal IACUC protocols have been approved by the University of Kansas Institutional Animal Care and Use Committee with protocol number 258-01 and operated in the KU Animal Care Unit.

Note that full information on the approval of the study protocol must also be provided in the manuscript.
